# Developmental or evaluative? Understanding the impact of algorithmic evaluation on gig workers’ thriving at work

**DOI:** 10.3389/fpsyg.2026.1899280

**Published:** 2026-08-18

**Authors:** Xinyu Teng, Huan Tao, Chuntong Dong

**Affiliations:** 1 School of Management, Huazhong University of Science and Technology, Wuhan, China; 2 School of Civil Engineering and Transportation, Northeast Forestry University, Harbin, China

**Keywords:** developmental algorithmic evaluation, evaluative algorithmic evaluation, gig workers, job crafting, SEM-ANN-NCA, thriving at work

## Abstract

Algorithmic evaluation plays a pivotal role in regulating gig workers’ job performance; however, extant studies have reported inconsistent findings regarding its effects. Although prior research has examined the consequences of algorithmic evaluation, limited attention has been paid to how it influences gig workers’ proactive behaviors such as job crafting, and subsequent thriving at work. To address this gap, this study identifies two types of algorithmic evaluation, namely developmental and evaluative algorithmic evaluation, and draws on approach-avoidance motivation theory to investigate their differential effects on thriving at work. Using survey data from 435 valid responses and employing an SEM-ANN-NCA approach, we find that evaluative algorithmic evaluation induces avoidance job crafting, which subsequently undermines thriving at work. In contrast, developmental algorithmic evaluation fosters approach job crafting, thereby enhancing thriving at work. Furthermore, time pressure strengthens the positive relationship between evaluative algorithmic evaluation and avoidance job crafting. This study contributes to the algorithmic management literature by revealing the dual nature of algorithmic evaluation and demonstrating how different evaluation approaches trigger distinct proactive behavioral responses and influence gig workers’ thriving at work.

## 1 Introduction

The rapid expansion of the gig economy has created numerous job opportunities while significantly advancing social welfare (Meituan Research Institute, 2020). For instance, Statista reported that, in 2020, the number of platform-based gig workers in the United States reached 59 million (Yu et al., 2025), whereas China represented an even larger gig labor market, with an estimated 175 million workers relying on gig platforms by 2024 (Gongmao New Economy Research Center, 2025). These figures suggest that platform-based gig employment has become a significant component of the labor market. Although the gig economy generates substantial social welfare, it is undeniable that gig workers frequently encounter serious psychological and physical health risks, such as perceived deprivation (Yu et al., 2025), and occupational burnout (Liu et al., 2026), ultimately making it difficult for them to sustain long-term work vitality (Yu et al., 2025). For example, prior research shows that 73.26% of food delivery riders in China leave their jobs within 1 year (Li and Jiang, 2022). In this regard, scholars have argued that these serious issues are largely driven by the pressures exerted on gig workers through platforms’ algorithmic evaluation mechanisms (Zhang et al., 2024; Li et al., 2025; Rahman, 2021; Kellogg et al., 2020).

In the context of gig work, gig platforms leverage depersonalized algorithmic evaluation mechanisms to monitor worker performance, thereby determine their eligibility for work, administering rewards and penalties, and even terminating workers (Möhlmann et al., 2021). However, extant literature has largely focused on the adverse effects of algorithmic evaluation on gig workers’ job outcomes (Zhang et al., 2024; Li et al., 2025), paying limited attention to how such evaluations may foster gig workers’ thriving at work to support their long-term platform careers. This gap deserves scholarly attention, as scholars have emphasized that enhancing gig workers’ thriving at work is critical to sustaining their long-term participation on gig platforms (Shi et al., 2024).

Thriving at work is conceptualized as a positive psychological state encompassing individual learning and vitality (Spreitzer et al., 2005). Compared with relatively static constructs such as job satisfaction and burnout, thriving at work captures a dynamic, forward-looking state of vitality and learning that more fully reflects gig workers’ ongoing self-development and long-term career sustainability (Spreitzer et al., 2005; Ashford et al., 2018; Kleine et al., 2019). A substantial body of literature has shown that thriving at work helps gig workers overcome the negative effects of work-related pressure (Ashford et al., 2018; Kleine et al., 2019), leading to positive job outcomes such as career adaptability, favorable job attitudes, and increased job performance (Jiang, 2017; Taneva and Arnold, 2018; Kleine et al., 2019). However, research on how algorithmic evaluation affects gig workers’ thriving at work remains scarce, potentially hindering platforms’ efforts to foster supportive work environments.

Furthermore, existing research on the impact of algorithmic evaluation on gig workers’ thriving at work faces several important limitations. On the one hand, the impersonal and automated nature of algorithmic evaluation leaves gig workers with little scope to modify evaluation rules, making volitional job crafting behaviors a vital adaptive mechanism (Weber et al., 2026; Shi et al., 2024). Consistent with this view, research on thriving at work suggests that thriving does not arise passively but is closely associated with workers’ proactive and agentic behaviors, particularly job crafting (Spreitzer et al., 2005). As a self-targeted proactive behavior, job crafting reflects workers’ conscious efforts to modify their work and alleviate work-related stress, with these efforts driven by distinct motivational orientations, including approach motivations aimed at pursuing desired outcomes and avoidance motivations aimed at preventing negative consequences (Ferris et al., 2011; Bruning and Campion, 2018; Ding et al., 2025). However, extant studies have largely framed gig workers’ responses to algorithmic evaluation as a dichotomy between compliance and resistance (Weber et al., 2026), overlooking how workers may proactively modify their work through job crafting and thereby shape their thriving at work. On the other hand, extant research regarding the effects of algorithmic evaluations on gig workers has yielded mixed findings. Consistent with the aforementioned practical concerns, most prior research found that algorithmic evaluations have negative psychological impacts, such as anxiety and frustration, since the algorithmic evaluation results directly affect gig workers’ job security, task opportunities, and income stability (Kellogg et al., 2020; Bellesia et al., 2023; Rahman, 2021). Conversely, other studies found that algorithmic evaluations produce positive psychological outcomes. For example, gig workers view algorithmic evaluations as a challenge to job demands (Li et al., 2025) and improve their skill through the clear and timely feedback provided by algorithmic evaluations (Zhang et al., 2024).

To reconcile this inconsistency, we propose that the inconclusive findings regarding the effects of algorithmic evaluation on gig workers arise from prior studies’ predominant reliance on unidimensional measures of algorithmic evaluation. Indeed, performance appraisal is inherently dualistic, encompassing both developmental and evaluative functions contingent on their underlying purposes (Meyer et al., 1981). Based on previous literature about performance evaluation (Boswell and Boudreau, 2000; Eyoun et al., 2020; Parent-Rocheleau et al., 2024), we propose that there are two kinds of algorithmic evaluations: developmental algorithmic evaluations and evaluative algorithmic evaluations. In details, developmental algorithmic evaluations mean that the evaluation results are used to provide performance feedback, identify workers’ strengths and weaknesses, and support professional skill development (Boswell and Boudreau, 2000; Eyoun et al., 2020), while evaluative algorithmic evaluations mean that evaluation results are used to guide personnel decisions such as task assignment, salary, and termination decisions (Möhlmann et al., 2021). Compared with traditional performance appraisal, algorithmic evaluation enables more comprehensive, immediate, and automated performance assessments (Kellogg et al., 2020). In this regard, this study posits that developmental algorithmic evaluation, characterized by timely and objective work feedback manifested through task completion metrics, progress bars, and gamified achievement badges, thereby supporting gig workers’ development by helping them identify performance gaps that can be narrowed through efforts within their capacity (Zhang et al., 2024). Conversely, evaluative algorithmic evaluation is closely linked to workers’ income and involves the continuous monitoring of indicators such as order rejection rates, customer cancellation rates, and late-delivery rates (Liu et al., 2026; Peng et al., 2025; Zhang et al., 2026), as well as asymmetric customer evaluations (Rahman, 2021), which may alert workers to potential changes in work priority and the threat of penalties.

Driven by these gaps above, this study aims to investigate how developmental and evaluative algorithmic evaluations influence thriving at work through job crafting. To address this question, the study draws on approach-avoidance motivation theory (AAMT). AAMT is particularly appropriate for our research context because it highlights the dual pathways of different individual proactive motivation orientations, whereby environmental stimuli can orient individuals toward either approaching desirable outcomes or avoiding undesirable outcomes (Ferris et al., 2011; Bruning and Campion, 2018). This dual-pathway perspective closely aligns with our research purpose. Specifically, AAMT suggests that developmental algorithmic evaluation can be interpreted as a challenging job demand that motivates gig workers to engage in approach job crafting to achieve desirable outcomes (such as improving performance rankings and increasing earnings). In contrast, evaluative algorithmic evaluation may be perceived as a threatening job demand that elicits avoidance job crafting to prevent undesirable outcomes (Elliot, 1999), such as narrowing work boundaries.

Moreover, AAMT posits that contextual stimulus can shape individuals’ motivational orientations and subsequent job crafting behaviors (Bruning and Campion, 2018). In our context, algorithmic evaluations prioritize on-time delivery (Alrashidi et al., 2026), and the piece-rate compensation structure incentivizes gig workers to maximize efficiency to sustain their livelihoods (Wang and Churchill, 2025). Thus, time pressure represents an institutionalized job demand that creates persistent psychological strain and constrains workers’ agency (Quy Nguyen-Phuoc et al., 2023). Accordingly, we propose that time pressure suppresses workers’ approach motivation to use developmental algorithmic evaluation in pursuing desirable outcomes, while strengthening their avoidance motivation by heightening the perceived threat of penalties associated with evaluative algorithmic evaluation. As such, we identify time pressure as a key boundary condition which may shape gig workers’ job crafting orientations. As such, the second aim of this study is to investigate (2) how time pressure moderates these relationships. To address these research questions, this study develops a context-specific theoretical model based on AAMT and empirically tests it using survey data.

This study makes the following theoretical contributions. First, this study extends the algorithmic management literature by identifying two distinct forms of algorithmic evaluation, namely developmental and evaluative algorithmic evaluation, thereby providing a more nuanced understanding of the heterogeneous nature of algorithmic evaluation. Second, by examining how these two forms of algorithmic evaluation influence gig workers’ thriving at work, this study extends existing algorithmic evaluation research by revealing an important yet underexplored outcome of algorithmic evaluation. Third, this study contributes to AAMT by extending its application to the algorithmic management context and demonstrating how algorithmic evaluation serves as a job demand that triggers different motivational orientations and job crafting behaviors. Meanwhile, this study identifies time pressure as an important boundary condition that extends our understanding of the effects of algorithmic evaluation on job crafting behaviors. Finally, by showing that algorithmic evaluation channels worker agency into adaptive job crafting through approach and avoidance motivation, this study refines the compliance-resistance binary framework.

## 2 Literature review and theoretical background

### 2.1 Gig workers’ thriving at work

Thriving at work is a positive psychological state characterized by a sense of learning and vitality (Spreitzer et al., 2005). Specifically, vitality refers to a positive sense of possessing energy and feeling “alive,” reflecting the hedonic dimension of psychological well-being (Spreitzer et al., 2005). Learning refers to workers’ perception that they are acquiring and applying knowledge or skills, representing the eudaimonic dimension of psychological well-being (Spreitzer et al., 2005). Unlike static constructs such as job satisfaction and burnout, thriving at work foregrounds the forward momentum arising from proactive learning and sustained vitality, thereby encapsulating individuals’ dynamic self-growth in the workplace (Goh et al., 2021). Prior research has shown that thriving at work not only enhances individuals’ physical and psychological well-being (Spreitzer et al., 2005; Walumbwa et al., 2018; Kleine et al., 2019), but also contributes to a range of positive work outcomes, such as greater career adaptability, more positive job attitudes, and improved job performance (Jiang, 2017; Taneva and Arnold, 2018; Kleine et al., 2019). Therefore, thriving at work has become an important topic in organizational behavior and workplace well-being research.

Most existing studies have examined the antecedents of thriving at work in traditional organizational settings, emphasizing the important roles of feedback, coworker support, supervisor support, and organizational resources. For example, prior research suggests that work-related resources, such as feedback from others, are particularly important for fostering thriving because they provide employees with valuable information for improvement, thereby promoting learning and vitality (Quinn and Dutton, 2005; Spreitzer et al., 2005). In addition, workplace contextual factors have been shown to play important roles in fostering employees’ thriving at work. For instance, coworker and supervisor support can help employees acquire new knowledge and facilitate personal growth (Colbert et al., 2016; Taneva and Arnold, 2018).

With the rapid development of the gig economy, research on thriving at work has begun to extend beyond traditional organizational settings to algorithmic platform-based contexts. A small but growing body of research has examined how gig workers’ agency contributes to thriving at work. For example, Mao et al. (2024), drawing on goal-setting theory, examined how gig workers’ self-leadership promotes thriving at work through goal clarity and goal commitment, and further found that perceived algorithmic autonomy support moderates this effect. Based on the proactive motivation model and work design literature, Shi et al. (2024) investigated how perceived algorithmic control influences gig workers’ thriving at work and found that perceived algorithmic control promotes thriving through job crafting, with job autonomy strengthening this indirect effect. Collectively, these studies suggest that gig workers are not merely passive recipients of platform-based algorithmic management; rather, their proactive agency and perceptions of the algorithmic environment can shape their learning and vitality.

However, the underlying psychological and behavioral mechanisms through which gig workers develop thriving at work remain insufficiently explained. Unlike employees in traditional organizations, gig workers typically complete platform tasks independently and have limited access to stable coworker interactions, supervisory feedback, and organizational support. Consequently, their performance feedback and behavioral adjustments are increasingly shaped by platform algorithms. Because algorithmic evaluation provides gig workers with real-time performance information and shapes their interpretations of performance expectations, improvement opportunities, and platform rules, it may serve as an important contextual factor influencing their learning and vitality. Although existing studies have explained the formation of gig workers’ thriving at work through the lenses of algorithmic control and algorithmic autonomy support, relatively little attention has been paid to algorithmic evaluation as a distinct performance feedback mechanism. Given that thriving at work enables gig workers to cope with persistent work pressures and maintain a positive work state (Ashford et al., 2018), examining how algorithmic evaluation influences gig workers’ thriving at work is of considerable theoretical and practical significance.

### 2.2 The impact of algorithmic evaluation on gig workers

In recent years, scholars have paid increasing attention to algorithmic evaluation. Traditional performance evaluation is typically conducted by organizational managers and primarily serves to review employees’ work activities, assess performance outcomes, and provide performance-related feedback (Kellogg et al., 2020). In contrast, performance assessment in the context of algorithmic evaluation relies increasingly on automated and data-driven algorithmic systems. Algorithmic evaluation automatically collects performance-related information about gig workers from internal platform operation data and external customer feedback, and then integrates and analyzes such information through algorithmic systems (Kellogg et al., 2020; Möhlmann et al., 2021). The primary functions of algorithmic evaluation include guiding workers toward platform-defined behaviors and supporting decisions related to compensation, task allocation, incentives, and other work-related outcomes (Rahman, 2021; Parent-Rocheleau et al., 2024; Zhang et al., 2024).

Existing research has shown that algorithmic evaluation has important implications for gig workers’ work experiences, behavioral responses, and other work outcomes. Table 1 summarizes empirical studies examining the consequences of algorithmic evaluation. As shown in Table 1, prior research has yielded mixed findings regarding the effects of algorithmic evaluation. On the one hand, some studies emphasize the controlling and stressful nature of algorithmic evaluation. Because evaluation outcomes are closely tied to task opportunities, income stability, platform ratings, and work eligibility, gig workers may experience negative psychological reactions, such as anxiety, helplessness, uneasiness, and frustration (Rahman, 2021; Bellesia et al., 2023). On the other hand, other studies suggest that algorithmic evaluation does not necessarily generate negative consequences. Timely, quantitative, and continuous algorithmic evaluation can help workers understand their service performance, identify areas for improvement, adjust work strategies, and enhance service performance (Quy Nguyen-Phuoc et al., 2023; Zhang et al., 2024). For example, Zhang et al. (2024) found that perceived algorithmic evaluation enhances app-workers’ service performance by increasing their flow experience. Taken together, these studies suggest that algorithmic evaluation can be either controlling or supportive, depending on its underlying purpose and workers’ interpretations.

**Table 1:** Summary of studies about the consequences of algorithmic evaluations.

Article	Algorithmic evaluation purposes		Method	Dependent variables	Theoretical lens
	Evaluative	Developmental			
Bellesia et al. (2023)	√	√	Interviews	Behavioural and emotional responses	Technology affordances theory
Salmon et al. (2025)	√		Survey	Job satisfaction	Organizational justice theory and organizational support theory
Li et al. (2025)	√		Survey	Work disengagement	Job demands-resources model
Peng et al. (2025)	√		Survey	Job burnout	Self-determination theory
Rahman (2021)	√		Interviews	Behavioral and emotional responses to evaluation opacity	Literature about third-party evaluations
Zhang et al. (2021)	√		Content analysis	Worker perceptions of HRM activities	Literature about human resource management (HRM) activities
Zhang et al. (2024)		√	Survey	Service performance	Flow theory and conservation of resources theory

Traditional performance appraisal research provides an important theoretical foundation for understanding the heterogeneous roles of algorithmic evaluation. In this regard, Meyer et al. (1981) argued that performance appraisal has a dual nature, serving developmental and evaluative purposes rather than representing a homogeneous managerial practice. Developmental performance evaluation emphasizes feedback, skill enhancement, and employee development, whereas evaluative performance evaluation focuses on performance judgment, outcome comparison, and managerial decision-making (Meyer et al., 1981). On algorithmic platforms, the traditional “manager-employee” evaluative relationship has increasingly been displaced by algorithmic technologies that continuously record, quantify, and evaluate workers’ performance in an automated manner (Kellogg et al., 2020). Crucially, the purpose-based distinction between developmental and evaluative evaluation remains theoretically valid for algorithmic contexts, because it rests on how evaluation information is used, rather than on whether the evaluator is a human manager or an algorithmic system. Therefore, this technological shift does not eliminate the distinction between developmental and evaluative purposes; rather, it reconfigures how these purposes are enacted.

In this study, developmental algorithmic evaluation refers to gig workers’ perception that algorithmic evaluation serves to provide developmental feedback and support their future performance improvement. Comparing with traditional performance evaluation, developmental algorithmic evaluation is characterized by the real-time and automated quantification of gig worker’ work performance, enabling platforms to provide dynamic and objective feedback on key performance indicators, such as completed orders, on-time delivery performance, and ranking changes (Zhang et al., 2024, 2026). To strengthen its motivational effects, platforms often incorporate gamified feedback mechanisms, such as digital points, task progress bars, achievement badges, and competitive leaderboards featuring titles such as “Strongest Hero” (Zhang et al., 2024). These mechanisms provide workers with clear performance feedback and encourage sustained effort by linking successful challenge completion to additional financial rewards and priority access to future tasks, while ensuring that unsuccessful attempts do not result in pay deductions (Bellesia et al., 2023).

By contrast, evaluative algorithmic evaluation refers to gig workers’ perception that algorithmic evaluation primarily serves evaluative purposes by informing employment-related decisions, such as performance sanctions, reduced eligibility for task allocation, and employment continuation. Due to its strong linkage with income and employment-related outcomes, evaluative algorithmic evaluation is more likely to be perceived as algorithmic control rather than developmental support (Liu et al., 2026). Specifically, it is characterized by the automated tracking of worker behaviors and the algorithmic scoring of their performance indicators, including order rejection rates, customer cancellation rates, excessive idle time, and late delivery rates (Peng et al., 2025; Liu et al., 2026; Zhang et al., 2026), while also incorporating asymmetric third-party customer evaluations into workers’ service evaluations (Rahman, 2021). Notably, prior research implies that the adverse effects of algorithmic evaluation on gig workers primarily stem from its evaluative function, and are further exacerbated by algorithmic features such as automated sanctioning, opacity, asymmetric customer evaluations, and limited negotiability. For example, algorithmic opacity limits workers’ understanding of how evaluation criteria and penalties are determined and weakens their ability to challenge algorithmic decisions (Li et al., 2025). Moreover, asymmetric customer evaluations may heighten gig workers’ concerns about negative reviews and compel them to provide time-consuming services beyond their formal role requirements (Rahman, 2021; Peng et al., 2025). In particular, automated sanctions are immediately enforced with little scope for workers’ contestation, and the lack of effective appeal channels may further intensify gig workers’ feelings of deprivation and powerlessness (Yu et al., 2025).

Drawing on this distinction, this study reviews existing algorithmic evaluation research from the perspective of evaluation purpose and identifies the evaluative functions examined in prior studies, as summarized in Table 1. However, as shown in Table 1, most existing quantitative studies conceptualize algorithmic evaluation as a unidimensional construct and rarely distinguish among its different evaluative purposes. This approach fails to capture the heterogeneous nature of algorithmic evaluation and limits our understanding of its differential effects on gig workers. Therefore, drawing on performance appraisal research, this study distinguishes between two forms of algorithmic evaluation: developmental algorithmic evaluation and evaluative algorithmic evaluation. By differentiating these two forms, this study further examines how distinct evaluative purposes shape gig workers’ thriving at work, thereby addressing the limitations of treating algorithmic evaluation as a single construct.

### 2.3 Approach-avoidance motivation theory

Approach-Avoidance Motivation Theory (AAMT) provides a useful theoretical lens for understanding individuals’ proactive motivational orientations and subsequent behavioral responses. Rooted in the classical distinction between approach and avoidance motivation in motivational psychology, AAMT has been widely adopted in organizational and management research to explain proactive work behaviors. Specifically, AAMT proposes that proactive work behaviors, such as job crafting, are driven by two distinct motivational orientations, namely approach and avoidance motivation (Elliot et al., 2006; Ding et al., 2025; Shang et al., 2023; Wang et al., 2025). These two motivational orientations represent fundamental yet opposing self-regulatory orientations: approach motivation directs individuals toward rewards, growth, and other desirable outcomes, whereas avoidance motivation drives individuals to prevent failure, pain, and other undesirable outcomes (Elliot and Thrash, 2010; Ferris et al., 2011).

Job crafting refers to the self-initiated and proactive changes that employees make to their jobs to improve their work experiences (Bruning and Campion, 2018). In organizational behavior research, job crafting has been recognized as an important form of proactive self-management that enables employees to adapt their work environments, enhance performance, and maintain psychological well-being (Bruning and Campion, 2018). Building on distinct motivational orientations, AAMT proposes that job crafting behaviors are driven by either approach motivation or avoidance motivation, resulting in two corresponding forms of job crafting: approach job crafting and avoidance job crafting (Bipp and Demerouti, 2015; Bruning and Campion, 2018). Specifically, approach job crafting involves proactive and effortful behaviors aimed at improving work by acquiring additional resources, expanding work roles and social interactions, and optimizing work organization (Bruning and Campion, 2018). In contrast, avoidance job crafting involves prevention-oriented behaviors aimed at reducing or avoiding work demands by minimizing hindering tasks, limiting social interactions, or withdrawing from certain work responsibilities (Bruning and Campion, 2018). Prior research further suggests that approach job crafting is generally associated with more favorable work outcomes, whereas avoidance job crafting is more strongly related to withdrawal behaviors and demonstrates weaker positive effects on employee outcomes (Bruning and Campion, 2018). Whereas other motivational theories, such as self-determination theory primarily emphasize how environmental factors facilitate or constrain individuals’ levels of autonomous motivation (Gagné and Deci, 2005), AAMT conceptualizes individuals as volitional actors whose motivational orientations are shaped by situational cues or stimuli (Elliot and Thrash, 2010; Bruning and Campion, 2018). In other words, AAMT is concerned with the direction of individuals’ motivational orientations (i.e., approach or. avoidance), not with the intensity or degree of their autonomous motivation. Moreover, Bruning and Campion (2018) emphasized that their approach-avoidance framework of job crafting draws on transactional theories of stress, which posit that individuals appraise work demands either as challenges to confront or as threats to avoid.

Turning to the context of this study, although gig workers operate outside traditional organizational settings, their daily activities remain continuously shaped by platform task rules, algorithmic evaluation standards, and customer interactions. From a job crafting perspective, gig workers may proactively adjust their work behaviors in response to algorithmic evaluation to improve performance, optimize service delivery, or reduce the risk of negative evaluations. Consistent with this view, emerging research suggests that algorithmic management can stimulate gig workers’ job crafting behaviors (Ding et al., 2025). For example, Liu and Yin (2024) found that algorithmic management may foster gig workers’ approach job crafting through gamified experiences, while simultaneously eliciting avoidance job crafting by reducing perceived job autonomy. Thus, AAMT provides an appropriate theoretical foundation for examining how algorithmic evaluation influences gig workers’ distinct job crafting behaviors.

Moreover, performance appraisal research suggests that appraisal purposes shape employees’ interpretations of evaluations and their subsequent attitudes and behavioral responses (Boswell and Boudreau, 2002; Eyoun et al., 2020). Prior research on algorithmic management has typically conceptualized algorithmic evaluation as a job demand imposed on workers (Yu et al., 2025; Liu et al., 2026; Zhang et al., 2026). Drawing on AAMT, algorithmic evaluation is more likely to be interpreted as a challenging job demand when it provides feedback and identifies opportunities for performance improvement (Bruning and Campion, 2018; Zhang et al., 2026). By contrast, when algorithmic evaluation is used for task allocation, income determination, sanctions, or eligibility control, it is more likely to be perceived as a threatening and hindering job demand (Rahman, 2021; Bellesia et al., 2023). Accordingly, approach and avoidance job crafting represent two proactive adjustment pathways that gig workers may adopt when responding to different purposes of algorithmic evaluation. These differentiated behavioral responses further explain how algorithmic evaluation influences gig workers’ learning and vitality. Therefore, this study treats approach and avoidance job crafting as mediating mechanisms linking developmental and evaluative algorithmic evaluation to thriving at work.

## 3 Research model and hypotheses development

**Figure 1:**
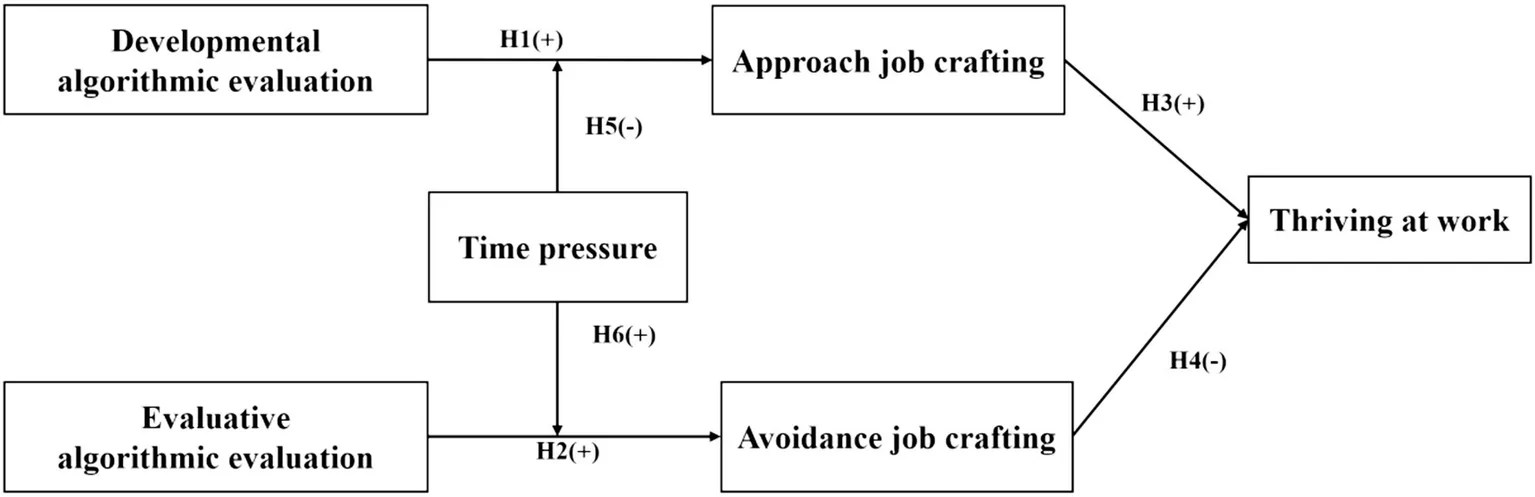
Research model.

Figure 1 presents the proposed research model. This research model aims to explain how developmental and evaluative algorithmic evaluation affects gig workers’ thriving at work.

According to AAMT, developmental algorithmic evaluation provides gig workers with feedback on their strengths and weaknesses to support future improvement (Roberts, 1995) Therefore, it can be conceptualized as a challenging job demand that motivates gig workers to engage in approach job crafting (Elliot, 1999). On the one hand, continuous algorithmic tracking provides gig workers with dynamic performance information such as digital points, task progress bars, and gamified badges, which serves as valuable job resources (Quy Nguyen-Phuoc et al., 2023; Wiener et al., 2023). By this way, developmental algorithmic evaluation provides objective feedback and guidance that helps gig workers identify areas for improvement and directs their efforts toward attainable better goals (Bellesia et al., 2023; Zhang et al., 2024). Meanwhile, these developmental cues activate the goal-achievement motive, prompting gig workers to proactively interpret and leverage algorithm-driven rules (Shi et al., 2024)., thereby fostering approach job crafting behaviors that are deliberately directed toward securing valued outcomes, such as increased earnings (Harju et al., 2016). Ultimately, favorable interactions between workers and algorithms facilitate the internalization of intrinsic work motivation, which in turn promotes long-term positive job crafting behavior (Shi et al., 2024). On the other hand, clear and quantified feedback enhances perceived algorithmic transparency (Zhang et al., 2024), and strengthens workers’ sense of control over performance improvement (Li et al., 2025). For example, workers can clearly understand which behaviors are valued and rewarded by the platform as indicators of high performance. By reducing uncertainty about platform performance goals, developmental algorithmic evaluation enables workers to proactively align their behaviors with algorithmic criteria through continuous, self-directed skill development, thereby fostering approach job crafting (Bruning and Campion, 2018). Therefore, we hypothesize that:

H1: Developmental algorithmic evaluation positively influences gig workers' approach job crafting.

Drawing on AAMT, evaluative algorithmic evaluation, which continuously and automatically quantifies workers’ performance to determine income- and employment-related outcomes, may be perceived as a threatening job demand that triggers avoidance job crafting (Elliot, 1999). First, because algorithmic evaluation outcomes directly affect gig workers’ livelihoods (Huang, 2023), the real-time tracking inherent in such evaluation may leave workers feeling continuously exposed to the threat of automated sanctions (Rahman, 2021). Coupled with the lack of effective appeal mechanisms, such evaluation practices may place gig workers in a disadvantaged position, thereby heightening their perceived deprivation (Yu et al., 2025; Zhao et al., 2026). Accordingly, workers are more likely to adopt cautious, risk-avoidant behaviors when confronted with uncertain or high-risk tasks to avoid adverse consequences for their algorithmic ratings. For example, when dealing with demanding customers or anticipating negative reviews, gig workers may narrow their job boundaries and avoid high-risk tasks by canceling orders, thereby reducing their exposure to unfavorable evaluations and performance-score deductions (Cameron and Rahman, 2022; Cameron, 2024). Second, prior studies indicate that algorithmic evaluation continuously monitors and assesses gig workers in real time to constrain their behavior, thereby reducing their autonomy and voice (Cram et al., 2022; Huang, 2023). In this context, gig workers may perceive platforms as treating them primarily as instruments for task completion rather than as workers whose interests are considered (Yu et al., 2025). This perceived loss of control heightens workers’ perceptions of task-related risks, prompting them to narrow their job boundaries by avoiding tasks likely to result in negative algorithmic evaluations (Bellesia et al., 2023; Cameron, 2024). Therefore, we have the following hypothesis:

H2: Evaluative algorithmic evaluation positively influences gig workers' avoidance job crafting.

Thriving at work refers to an individual’s experience of vitality and learning (Spreitzer et al., 2005). According to the AAMT, approach motivation, such as approach job crafting, is associated with positive emotions, attitudes, and proactive behaviors (Elliot, 1999; Elliot et al., 2006). Therefore, the approach job crafting could positively affect thriving at work. There are several reasons. First, the approach job crafting enables workers to build and expand social resources (Holman et al., 2024), which could foster job-related knowledge acquisition. For example, gig workers could actively seek and share information from gig platform communities, which help them improve their work performance (Zhang et al., 2025). This information serves as an important knowledge resource to promote thriving at work (Spreitzer et al., 2005). Second, the approach job crafting encourages gig workers to continuously learn new skills (Decius et al., 2023), which could make individuals develop a sense of learning as a result of their improved knowledge and skills (Mansour and Tremblay, 2020). Additionally, by proactive learning new skills, gig workers can experience a sense of autonomy, which further enhances workers’ positive affective experience and promotes their vitality (Bakker and Oerlemans, 2019). Finally, workers engaged in approach job crafting are typically more future-oriented and seeking more challenges in their work (Bruning and Campion, 2018; Bindl et al., 2019). By embracing new challenges, gig workers are motivated to continuously learn and apply new knowledge, resulting in an experience of learning (Prem et al., 2017). Therefore, we hypothesize that:

H3: Approach job crafting positively influences thriving at work.

Conversely, avoidance job crafting, which involves avoiding challenges and narrowing the work boundary (Bruning and Campion, 2018), negatively affects thriving at work. According to the AAMT, avoidance motivation, such as avoidance job crafting, is associated with negative emotions, attitudes, and proactive behaviors (Elliot, 1999; Elliot et al., 2006). Therefore, avoidance job crafting, which involves avoiding challenges and narrowing the work boundary (Bruning and Campion, 2018), may negatively affect thriving at work. There are several reasons. First, while avoidance job crafting can help gig workers protect themselves from unfavorable aspects of their work, it also limits their learning opportunities. Specifically, avoidance job crafting involves a reduction of one’s work role, such as reductions in tasks and responsibilities (Bruning and Campion, 2018). These reductions limit gig workers’ opportunities for exposure to diverse tasks and optimal levels of job challenges (Petrou et al., 2018), which leads gig workers to miss out on opportunities to develop professional skills and personal growth. Therefore, this reduction in learning opportunities could undermine the development of gig workers’ thriving at work. Second, avoidance job crafting leads to a decline in work engagement (Lichtenthaler and Fischbach, 2019; Costantini, 2024), which in turn inhibits vitality and learning at work. Specifically, by reducing challenging job resources and job demands, avoidance job crafting could result in a low level of work engagement (Petrou et al., 2012), as it diminishes the motivation and sense of meaningfulness in the work of gig workers (Mäkikangas, 2018). Further, low levels of work engagement relates to fewer learning opportunities and reduced access to support, which further reduces gig workers’ thriving at work (Kleine et al., 2019). Based on these, we hypothesize that:

H4: Avoidance job crafting negatively influences thriving at work.

In the gig economy context, time pressure as a job demand is often associated with high-intensity tasks and tight deadlines (Quy Nguyen-Phuoc et al., 2023). We predict that the impact of developmental algorithmic evaluation on approach job crafting is moderated by perceived time pressure. First, under high time pressure, gig workers’ cognitive and behavioral resources are primarily directed toward addressing urgent tasks at hand, limiting their capacity to act on long-term improvement goals. In other words, time pressure limits workers’ ability to fully utilize developmental algorithmic evaluation, since their resources are fully occupied by meeting immediate deadlines. Second, high time pressure discourages workers from investing time in skill development, such as experimenting with new work strategies, since doing so could increase the likelihood of platform penalties for exceeding deadlines. This makes it harder for gig workers to balance short-term task demands with long-term improvement, thereby diminishing the positive effect of developmental algorithmic evaluation on approach job crafting. Therefore, we hypothesize that:

H5: Time pressure weakens the positive impact of developmental algorithmic evaluation on approach job crafting.

By contrast, we propose that the relationship between evaluative algorithmic evaluation and avoidance job crafting is moderated by perceived time pressure. High time pressure increases the likelihood of gig workers receiving negative algorithmic evaluations, such as penalties for exceeding deadlines. Since performance evaluations with evaluative purpose could affect income and platform eligibility (Huang, 2023), gig workers are more likely to employ avoidance job crafting under high time pressure to avoid undesirable consequences, such as negative evaluations. Previous studies have indicated that individuals facing adverse conditions are more likely to adopt avoidance job crafting as a coping strategy to protect themselves from loss (Petrou and Xanthopoulou, 2021; Lu et al., 2022). For instance, if a gig worker faces time pressure, they are more likely to avoid challenging tasks which is time-consuming to avoid the undesirable algorithmic evaluation results. Therefore, we hypothesize that:

H6: Time pressure strengthens the positive effect of evaluative algorithmic evaluation on avoidance job crafting.

## 4 Research design and methodology

### 4.1 Measurement development

The research model was examined with the survey method. We employed scales that have been validated in prior research and tailored to the specific context of our study. To guarantee the accuracy of the questionnaire items, this study introduced back-translation technique to translate the original English questionnaire into Chinese. Finally, we invited several scholars and gig workers to review the questionnaire for logical coherence, accuracy, and comprehensibility. The questionnaire employed 7-point Likert scales, with 1 being “strongly disagree,” 4 being “neutral,” and 7 being “strongly agree.” Table A-1 of Appendix A displays the scales.

### 4.2 Data collection

The formal survey was then distributed using Credamo (https://www.credamo.com). We took the following four measures to ensure the quality of the data. First, we selected food delivery workers as our participants by using the sample selection services of Credamo. Second, attention check items were embedded in the questionnaire. Those who failed in these checks were excluded. Finally, we filtered out samples that finished in an unreasonably short time (i.e., less than one minute) or selected the same option for all questions. Based on these criteria, a total of 435 valid responses were obtained. Table 2 displays the demographic information of the sample.

**Table 2:** Demographics of respondents (*N* = 435).

Demographic	Item	N	Percentage
Gender	Male	315	72.4%
	Female	120	27.6%
Age	Below 20	11	2.5%
	21–30	299	68.7%
	31–40	104	23.9%
	41–50	9	2.1%
	51–60	12	2.8%
Service duration	Below 3 months	9	2.1%
	3–6 months	37	8.5%
	6 months–1 years	77	17.7%
	1–2 years	160	36.8%
	Above 2 years	152	34.9%
Family status	Unmarried	220	50.6%
	Married without children	38	8.7%
	Married with children	177	40.7%
Income (per month)	Below 3,000	6	1.4%
	3,000–5,000	59	13.6%
	5,000–10,000	213	49.0%
	10,000–20,000	133	30.6%
	Above 20,000	24	5.4%

### 4.3 Data analysis

In this study, we introduced partial least squares structural equation modeling (PLS-SEM), which is a method that has been widely employed to empirically examine the antecedents that result in an outcome. Since both thriving at work and approach job crafting are higher-order constructs with reflective first-order and formative second-order structures, the PLS-SEM is a suitable choice. Moreover, although PLS-SEM is suitable for testing hypotheses, it can only examine the linear relationship between variables. However, since the relationships between individuals’ decisions/and behaviors are not always linear, PLS-SEM may lead to oversimplification or premature conclusions. This limitation of PLS-SEM could be complemented by a non-linear and artificial neural network (ANN) model, which could examine the nonlinearity relationships between variables (Arpaci and Bahari, 2023). Therefore, this study examined the proposed hypothesis with PLS-SEM and further validated our findings through ANN. Furthermore, we also adopted the necessary condition analysis (NCA), which is a novel approach to explore the necessary conditions in datasets (Dul et al., 2020), to further explore the necessary relationships between variables. The NCA. By adopting a hybrid SEM-ANN-NCA method, we could leverage the strengths of each methodology and complement each other (Loh et al., 2022).

#### 4.3.1 Common method bias

Since self-reported survey data were used in the present study, common method bias may be an issue. Thus, we performed a series of tests to evaluate common method variance. First, to reduce respondents’ cognitive biases, we randomized the sequence of items for the dependent and independent variables during the survey design (Podsakoff et al., 2003). Second, Harman’s single-factor test was performed (Podsakoff et al., 2003). The factor with the largest proportion explained a total variance of 34.156%, which is below the 40% threshold. Third, we incorporated a common technique factor into the model (Liang et al., 2007). The majority of the method factor loadings were not significant, and the average method variance only accounted for 0.56% of the actual variance (see Appendix Table A-2). Finally, we used the marker variable technique to do additional CMB testing. We chose perceived media richness as the marker variable and found that the relationships between Perceived media richness and approach job crafting, avoidance job crafting, thriving at work were all below the 0.3 threshold. After adding the marker variable, the significance of all hypotheses remained unchanged (Lindell and Whitney, 2001). In summary, the results of these tests show that common technique bias is not a threat in our study.

#### 4.3.2 Measurement model test

To make sure the measurements of constructs are valid and reliable, a number of tests have been conducted. First, the discriminant validity was tested with exploratory factor analysis. At first, the factor loadings for each construct were higher than 0.7 and the cross loadings for the other factors were lower than 0.5, indicating good discriminant validity of the scale (As presents in Appendix Table A-3). Next, we examined the reliability of each construct. The results indicated that the Cronbach’s *α* and Composite Reliability (CR) values for each construct were above 0.7, suggesting that all constructs have good reliability (Straub et al., 2004).

Moreover, we assessed the validity of each construct, focusing on both convergent and discriminant validity. As presented in Table 3, the average variance extracted (AVE) for all the construct exceeded the threshold of 0.5, indicating good convergent validity of the measurement model. Furthermore, as indicated in Table 4, the square root of the AVE for each construct was greater than the correlation coefficients between that construct and any other construct, confirming good discriminant validity. Table 5 represents the results for HTMT, and the value for each construct did not exceed the threshold of 0.9.

**Table 3:** Reliability and validity.

Factors	Factor loading	Cronbach’s *α*	CR	AVE
DPA	0.742	0.756	0.845	0.578
	0.781			
	0.792			
	0.723			
EPA	0.897	0.793	0.877	0.705
	0.816			
	0.804			
ICH	0.845	0.791	0.878	0.706
	0.834			
	0.841			
ISOR	0.855	0.814	0.890	0.731
	0.903			
	0.803			
ISTR	0.775	0.735	0.850	0.654
	0.812			
	0.838			
AVJC	0.915	0.904	0.939	0.837
	0.896			
	0.934			
LEA	0.858	0.788	0.876	0.702
	0.852			
	0.804			
VIT	0.912	0.886	0.929	0.814
	0.909			
	0.885			
TIP	0.802	0.799	0.881	0.712
	0.882			
	0.846			

DPA = developmental algorithmic evaluations; EPA = evaluative job crafting; ICH = increasing challenges; ISOR = increasing social resources; ISTR = increasing structural resources; AVJC = avoidance job crafting; LEA = learning; VIT = vitality; TIP = time pressure.

**Table 4:** Discriminant validity.

Construct	DPA	EPA	ICH	ISOR	ISTR	AVJC	LEA	VIT	TIP
DPA	0.760								
EPA	0.000	0.840							
ICH	0.476	−0.051	0.840						
ISOR	0.432	−0.020	0.400	0.855					
ISTR	0.480	−0.008	0.582	0.468	0.809				
AVJC	−0.335	0.274	−0.296	−0.307	−0.335	0.915			
LEA	0.549	−0.093	0.494	0.544	0.522	−0.321	0.838		
VIT	0.501	−0.099	0.471	0.442	0.501	−0.350	0.670	0.902	
TIP	0.535	−0.033	0.499	0.498	0.531	−0.377	0.652	0.573	0.844

**Table 5:** Heterotrait-monotrait ratio of correlations.

Construct	DPA	EPA	ICH	ISOR	ISTR	AVJC	LEA	VIT	TIP
DPA									
EPA	0.052								
ICH	0.610	0.063							
ISOR	0.549	0.056	0.496						
ISTR	0.644	0.039	0.762	0.601					
AVJC	0.397	0.313	0.349	0.347	0.409				
LEA	0.714	0.119	0.626	0.677	0.687	0.373			
VIT	0.616	0.118	0.562	0.518	0.621	0.379	0.801		
TIP	0.692	0.066	0.626	0.622	0.689	0.426	0.825	0.676	

Additionally, we validated the second-order constructs of thriving at work and approach job crafting (the results were shown in Appendix A-4). For the approach job crafting, increasing challenges (weight: 0.379), increasing social resources (weight: 0.458), and increasing structural resources (weight: 0.400) significantly influenced approach job crafting, highlighting their importance in its formation. The variance inflation factors (VIF) of its first-order construct were lower than the threshold of 3, indicating that multicollinearity is not a threat. Similarly, for thriving at work, the VIF for the first-order constructs of thriving at work were all below 3. Its’ indicators learning and vitality (with weight of 0.669 and 0.420, respectively) significantly influenced thriving at work.

#### 4.3.3 Hypotheses test

To test the hypotheses, PLS-SEM was performed using SmartPLS 4.1. The results suggest that developmental algorithmic evaluation positively affects approach job crafting (*b* = 0.308, *f*^2^ = 0.113, *p* < 0.001), which lends support to H1. Evaluative algorithmic evaluation (*b* = 0.223, *f*^2^ = 0.059, *p* < 0.001) positive affects avoidance job crafting, thus supporting H2. Additionally, approach job crafting (*b* = 0.631, *f*^2^ = 0.616, *p* < 0.001) significantly influences thriving at work, thus supporting H3. Avoidance job crafting (*b* = −0.114, *f*^2^ = 0.020, *p* < 0.01) negatively affect thriving at work, thus supporting H4.

**Figure 2:**
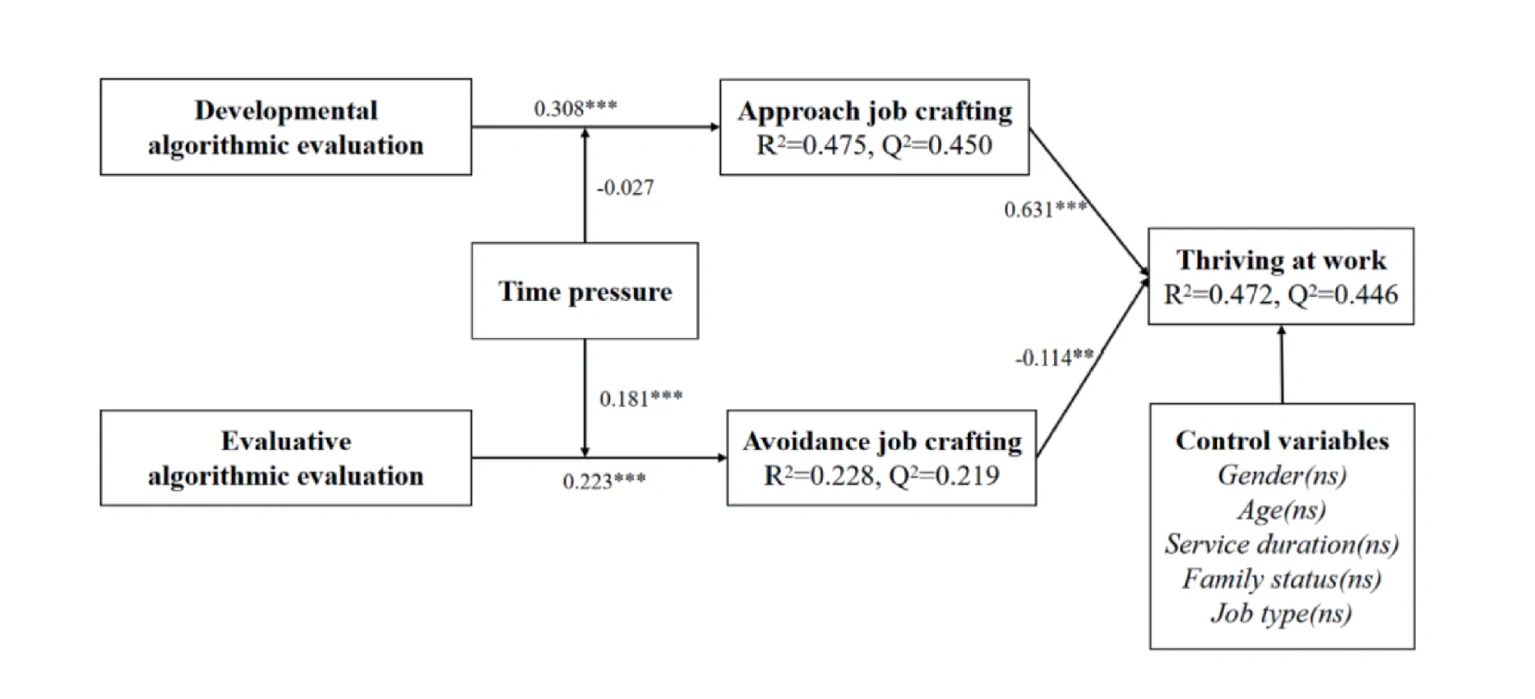
Results of hypotheses testing. ***p < 0.001, **p < 0.01, *p < 0.05, n.s. nonsignificant.

Regarding the moderating effect, time pressure does not have a significant moderating impact on the relationship between developmental algorithmic evaluation and approach job crafting (*b* = −0.027, *f*^2^ = 0.003, *p* = 0.469), failing to support H5. A possible explanation is that developmental algorithmic evaluation primarily focuses on employees’ long-term growth, which is less likely to be influenced by time pressure in the short term. Meanwhile, the results show that time pressure has a positive moderating effect on the relationship between evaluative algorithmic evaluation and avoidance job crafting (*b* = 0.181, *f*^2^ = 0.026, *p* < 0.001), thus supporting H6. Figure 2 presents the structural model estimation results that include the effects of control variables.

#### 4.3.4 Artificial neural network analysis

PLS-SEM was performed to examine the proposed theoretical model and hypotheses. Following the guidance of prior literature, ANN analysis was further conducted to complement the PLS-SEM results by examining complex relationships among variables through the connections between network layers (Chong, 2013; Leong et al., 2025). Based on the PLS-SEM results, the significant variables were used as inputs to the neural network. Consistent with previous studies (Chong, 2013; Basit et al., 2024; Leong et al., 2025), we tested neural network models with 1 to 10 hidden nodes.

For model 1, the inputs were developmental algorithmic evaluation, while the output was the approach job crafting. For model 2, the inputs were evaluative algorithmic evaluation and time pressure, while the output was avoidance job crafting. For model 3, the inputs were approach job crafting and avoidance job crafting, while the output was thriving at work. Meanwhile, this study also used the 10-fold cross-validation approach, where 90 percents of the survey data served as the training set and the remaining 10 percent was used to test the model’s prediction accuracy. The root means square error (RMSE) for both the training and test models is presented in Table 6. As shown, all the RMSEs are relatively low, indicating that the ANN model is reliable (Hew et al., 2018).

**Table 6:** RMSE for validation.

Network	Model 1		Model 2		Model 3	
	Training	Test	Training	Test	Training	Test
ANN1	0.529	0.494	0.639	0.523	0.524	0.588
ANN2	0.520	0.709	0.628	0.584	0.545	0.613
ANN3	0.530	0.510	0.623	0.599	0.566	0.536
ANN4	0.533	0.524	0.654	0.616	0.532	0.497
ANN5	0.551	0.508	0.617	0.589	0.562	0.390
ANN6	0.542	0.643	0.635	0.630	0.537	0.500
ANN7	0.538	0.355	0.626	0.566	0.547	0.506
ANN8	0.530	0.466	0.607	0.720	0.538	0.458
ANN9	0.527	0.421	0.632	0.564	0.547	0.462
ANN10	0.526	0.486	0.630	0.538	0.542	0.490
Average	0.532	0.512	0.629	0.593	0.544	0.504

Table 7 reports the outcomes of the sensitivity analysis, which examined the average contribution of input variables to the prediction of output variables across 10 neural networks. As presented in Table 9, developmental algorithmic evaluation holds the highest relative importance to predict approach job crafting, whereas time pressure holds the highest relative importance for predicting avoidance job crafting. Moreover, the approach to job crafting holds the highest relative importance for predicting thriving at work. These findings are in line with the findings of SEM, which further validate the robustness of our findings.

**Table 7:** Relative variable importance.

Network	Model 1	Model 2		Model 3	
	DPA	EPA	TIP	APJD	AVJC
ANN1	1	0.411	0.589	0.841	0.159
ANN2	1	0.478	0.522	0.82	0.18
ANN3	1	0.486	0.514	0.617	0.383
ANN4	1	0.243	0.757	0.856	0.144
ANN5	1	0.481	0.519	0.862	0.138
ANN6	1	0.499	0.501	0.89	0.11
ANN7	1	0.467	0.533	0.869	0.131
ANN8	1	0.403	0.597	0.84	0.16
ANN9	1	0.458	0.542	0.968	0.032
ANN10	1	0.487	0.513	0.873	0.127
Relative importance	1	0.467	0.533	0.844	0.156

#### 4.3.5 Necessary condition analysis

After examining the sufficient condition with PLS-SEM, the NCA is conducted to examine the necessity of all the relationships in the proposed research model. Table 8 presents the results of the NCA analysis. DPA, TIP, and APJD are the necessary conditions for thriving at work. Moreover, the bottleneck analysis results in Table 9 suggest that to reach a 100% level of approach job crafting, 82.5% level of DPA and 84.4% level of TIP are required. Similarly, to reach a 100% level of avoidance job crafting, 80.1% level of EPA and 76.9% level of TIP are required. To reach a 100% level of thriving at work, 63.6% level of APJD and 5.6% level of AVJC are required.

**Table 8:** Effect size of NCA.

Parameters	Index	Ceiling zone	Effect size	# above	c-accuracy	*p*-value	*p*-accuracy
DPA → APJD	CE-FDH	5.917	0.198	0	100%	0.000	0.002
	CR-FDH	7.516	0.251	8	98.2%	0.000	0.002
TIP→APJD	CE-FDH	5.852	0.190	0	100%	0.000	0.002
	CR-FDH	7.725	0.250	16	96.3%	0.000	0.002
EPA → AVJC	CE-FDH	13.889	0.386	0	100%	0.000	0.002
	CR-FDH	12.639	0.351	6	98.6%	0.000	0.002
TIP→AVJC	CE-FDH	3.778	0.111	0	100%	0.909	0.018
	CR-FDH	3.039	0.089	3	99.3%	0.882	0.020
APJD→TAW	CE-FDH	11.222	0.364	0	100%	0.000	0.002
	CR-FDH	9.138	0.296	4	99.1%	0.000	0.002
AVJC→TAW	CE-FDH	0.056	0.002	0	100%	0.747	0.027
	CR-FDH	0.028	0.000	0	100%	0.747	0.027

DPA = developmental algorithmic evaluations, EPA = evaluative algorithmic evaluations, AVJC = avoidance job crafting, APJD = approach job crafting, TIP = time pressure.

**Table 9:** The results of bottleneck analysis.

Outcome percent (%)	APJD		AVJC		TAW	
	DPA	TIP	EPA	TIP	APJD	AVJC
0	NN	NN	NN	NN	NN	NN
10	NN	NN	NN	NN	2.1	NN
20	NN	NN	7.0	NN	8.9	NN
30	NN	NN	16.1	NN	15.8	NN
40	1.2	NN	25.3	NN	22.6	NN
50	14.7	13.3	34.4	NN	29.5	NN
60	28.3	27.5	43.5	NN	36.3	NN
70	41.8	41.7	52.7	NN	43.1	NN
80	55.4	56.0	61.8	10.7	50.0	NN
90	68.9	70.2	71.0	43.8	56.8	NN
100	82.5	84.4	80.1	76.9	63.6	5.6

## 5 Discussion and implications

### 5.1 Discussion of the results

Given that thriving at work is important for gig workers to sustain their careers in high-pressure platform work environments (Ashford et al., 2018), this study draws on approach-avoidance motivation theory and uses survey data from 435 gig workers, analyzed through the SEM-ANN-NCA approach, to examine how developmental and evaluative algorithmic evaluations influence gig workers’ thriving at work through approach and avoidance job crafting. It also examines the boundary role of time pressure. This study does not treat algorithmic evaluation as a uniformly positive or negative factor. Instead, it distinguishes between its developmental and evaluative purposes and examines how these two types of algorithmic evaluation shape gig workers’ learning and vitality through different job crafting pathways. The findings provide a more nuanced perspective for explaining why prior studies have reported both positive and negative consequences of algorithmic evaluation.

First, the results show that developmental algorithmic evaluation positively affects approach job crafting, whereas evaluative algorithmic evaluation positively affects avoidance job crafting. This finding suggests that gig workers do not respond to algorithmic evaluation in the same way; rather, their behavioral responses depend on how they interpret the purpose of the evaluation. When algorithmic evaluation is perceived as developmental, it provides workers with information about their strengths, weaknesses, and directions for improvement, making them more likely to view evaluation as a resource for improving service, developing capabilities, and optimizing work strategies. For example, if food delivery workers receive specific feedback from algorithmic evaluation regarding delivery punctuality, customer communication, or service quality, they may be more likely to proactively adjust their order-taking strategies, communication approaches, or delivery arrangements. By contrast, when algorithmic evaluation is perceived as evaluative, workers are more likely to associate evaluation results with task opportunities, income stability, platform ratings, penalties, or work eligibility. In this case, algorithmic evaluation may be interpreted as a source of pressure or threat, leading workers to engage in avoidance job crafting to reduce potential risks. This finding is consistent with prior research on developmental and evaluative performance appraisal conducted by human managers, which suggests that different appraisal uses may lead to different employee attitudes and behavioral responses (Boswell and Boudreau, 2002). This study further shows that this logic also applies to platform-based algorithmic performance evaluation.

Second, the results indicate that approach job crafting positively affects thriving at work, whereas avoidance job crafting negatively affects thriving at work. This finding helps explain why gig workers’ proactive adjustments do not necessarily lead to positive psychological outcomes. Approach job crafting is oriented toward improvement, problem solving, and resource acquisition. It can create more learning opportunities for gig workers, enhance their sense of competence, and help them maintain vitality at work. For example, when gig workers proactively optimize service processes, learn more effective communication strategies, or improve task completion methods, they are more likely to experience learning and achievement at work, thereby maintaining higher levels of work energy. In contrast, avoidance job crafting is mainly oriented toward reducing undesirable demands or avoiding potential negative consequences. Although such behaviors may help workers reduce pressure in the short term, they may also limit their exposure to learning opportunities, weaken their active engagement with work tasks, and gradually reduce their positive energy at work. For example, if gig workers consistently protect their ratings by avoiding complex orders, reducing customer interactions, or lowering task involvement, their work experience may become increasingly narrowed and defensive, which is not conducive to sustained learning and vitality. Thus, the two types of job crafting represent two distinct behavioral pathways through which algorithmic evaluation shapes gig workers’ learning and vitality.

Finally, this study also reveals that time pressure plays different moderating roles in the relationships between developmental/evaluative algorithmic evaluation and approach/avoidance job crafting. Specifically, time pressure significantly strengthens the positive effect of evaluative algorithmic evaluation on avoidance job crafting. This suggests that time pressure makes the controlling and threatening implications of evaluative algorithmic evaluation more salient. When gig workers perceive that algorithmic evaluation results may affect their income, ratings, or platform eligibility, higher time pressure may further intensify their concerns about mistakes, delays, complaints, or low ratings. As a result, they are more likely to engage in avoidance job crafting, such as reducing exposure to high-risk tasks or avoiding situations that may lead to negative evaluations. Taking food delivery workers as an example, when delivery time is tight and evaluation results are linked to income or order allocation opportunities, workers may be more inclined to avoid orders involving complex routes, long waiting times, or demanding customers in order to reduce the risk of delays and negative ratings. This result suggests that time pressure is not merely a general job demand; rather, it may amplify the threatening attributes of evaluative algorithmic evaluation and make workers more likely to adopt defensive behavioral adjustments. In contrast, time pressure does not significantly moderate the relationship between developmental algorithmic evaluation and approach job crafting. This finding suggests that the positive effect of developmental algorithmic evaluation on approach job crafting remains robust across different levels of time pressure. Consistent with Pongsapan et al. (2026)‘s work, one possible explanation is that when gig workers possess sufficient work resources, time pressure is not necessarily perceived as detrimental. Rather, it can be internalized as a manageable and legitimate job demand that motivates gig workers to maintain a stable income (Pongsapan et al., 2026). In this context, workers’ approach motivation is activated, encouraging them to proactively engage in approach job crafting behaviors (e.g., optimizing delivery routes or refining order-assignment strategies) to better cope with time pressure. Consequently, the positive relationship between developmental algorithmic evaluation and approach job crafting remains unaffected by variations in time pressure.

### 5.2 Theoretical implications

This study has several theoretical implications. First, this study extends the algorithmic evaluation literature by distinguishing between two conceptually distinct forms of algorithmic evaluation. Prior research has generally conceptualized algorithmic evaluation as a unidimensional construct (Kellogg et al., 2020; Rahman, 2021; Bellesia et al., 2023), thereby limiting our understanding of its. This study differentiates between developmental and evaluative algorithmic evaluation. Our findings showed that evaluative algorithmic evaluation positively affects avoidance job crafting, whereas developmental algorithmic evaluation positively affects approach job crafting. In addition, time pressure only moderates the relationship between evaluative algorithmic evaluation and avoidance job crafting. These findings revealed that developmental and evaluative algorithmic evaluation exert different effects on gig workers. Taken together, this study contributes to the algorithmic evaluation literature by clarifying its distinct dimensions and uncovering their divergent effects, thereby providing a more nuanced understanding of the nature of algorithmic evaluation.

Second, this study advanced algorithmic evaluation literature by investigating how developmental and evaluative algorithmic evaluations affect gig workers’ thriving at work. Thriving at work is crucial for gig workers to maintain long-term and sustainable careers on digital labor platforms (Shi et al., 2024). Although prior studies have made admirable contributions to understanding the adverse effects of algorithmic evaluation on gig workers (Zhang et al., 2024; Li et al., 2025), relatively little attention has been paid to how algorithmic evaluation influences their thriving at work through self-targeted proactive behaviors such as job crafting. Job crafting is regarded as an adaptive, proactive response to work demands and is propelled by either approach or avoidance motivational orientation. Therefore, this study drew on AAMT and proposed a research model explaining how developmental and evaluative algorithmic evaluation influence gig workers’ thriving at work through distinct forms of job crafting. Our findings indicated that developmental algorithmic evaluation positively affects thriving at work through approach job crafting. Specifically, developmental algorithmic evaluation encourages gig workers to view algorithmically identified performance gaps as manageable challenges and opportunities for improvement. By signaling that greater effort can yield desired income outcomes, such feedback directs workers toward attainable goals and motivates them to adapt their work to algorithmic requirements, thereby promoting approach job crafting and enhancing their thriving at work. By contrast, evaluative algorithmic evaluation undermines workers’ thriving at work by eliciting avoidance job crafting. Because such evaluation is used to impose performance sanctions and determine continued access to work opportunities, workers are likely to interpret its feedback as a threat carrying potential losses in income and task access. Its impersonal and automated nature further exposes workers to persistent pressures over which they have limited control, prompting them to defensively reduce their exposure to algorithmic penalties through avoidance job crafting. These protective adjustments may restrict learning, diminish vitality, and ultimately impair workers’ thriving at work. Accordingly, this study extends current understanding of how algorithmic evaluation shapes gig workers’ thriving at work. In response to Weber et al. (2026), it also broadens the conceptualization of algoactivism by demonstrating that workers’ agency extends beyond compliance and resistance to include proactive adaptations through which they navigate job demands and safeguard their well-being.

Third, this study contributes to AAMT in two ways. On the one hand, although AAMT has often been used to explain work-related motivations and behaviors (Diefendorff and Mehta, 2007; Johnson et al., 2013; Aryee et al., 2017; Arain et al., 2019), its applicability in the context of algorithmic evaluation remains underexamined. This study extends the theory to the context of algorithmic management, thereby broadening its explanatory scope. On the other hand, this study extends the boundary conditions of AAMT. The results show that time pressure exerts asymmetric moderating effects on the two algorithmic evaluation pathways. It strengthened only the positive relationship between evaluative algorithmic evaluation and avoidance job crafting, without weakening the relationship between developmental algorithmic evaluation and approach job crafting. Prior research has largely viewed time pressure as a threatening job demand that depletes individual resources, inhibits positive work behaviors (Quy Nguyen-Phuoc et al., 2023). Within the theoretical framework of approach-avoidance motivation theory, this study extends this view by showing that the effects of time pressure are context dependent rather than inherently detrimental. These findings identify a contextual mechanism of algorithmic management through which time pressure can shift from a threatening to a manageable demand, thereby advancing the theoretical understanding of time pressure. Taken together, this study offers a new theoretical lens for understanding the differential effects of developmental and evaluative algorithmic evaluations on gig workers’ thriving at work.

### 5.3 Practical implications

This study also provides some practical implications. We advise platform managers to clearly distinguish between the developmental and evaluative dimensions of algorithmic evaluation, thereby safeguarding platform’s operational efficiency while cultivating thriving at work among gig workers. It is recommended that the developmental function of algorithmic evaluation be enhanced while its evaluative use be judiciously calibrated to maintain necessary managerial control.

First, this paper offers actionable guidance for platform managers to optimize developmental algorithmic evaluation systems. We suggest that platform managers incorporate motivational gamification elements into the evaluation process. For example, when gig workers meet predefined performance targets or unlock new tasks, platforms could provide immediate and visually engaging feedback through virtual avatars or achievement badges. Such gamified feedback could make the evaluation process more engaging and enjoyable, thereby fostering workers’ approach motivation to pursue personal achievement and engage in approach job crafting. Furthermore, developmental assessment processes combining supportive feedback with follow-up guidance enhance employee performance and job satisfaction (Engelbrecht and Fischer, 1995; Boswell and Boudreau, 2000). Accordingly, we suggest that developmental algorithmic evaluation should be further optimized by providing gig workers with dynamic, resource-based work guidance tailored to their ongoing progress. Specific optimization measures could include incorporating personalized guidance that directs workers to high-demand task areas based on their proficiency, recommendations for progressively more challenging tasks, and individualized pop-up notifications that recognize and encourage strong performance.

Second, platform managers should carefully calibrate the evaluative use of algorithmic evaluation to preserve platform operational efficiency without compromising gig workers’ well-being. Managers are advised to reduce the intensity of evaluative algorithmic evaluation by designing a more tolerant and flexible evaluation system. As a specific measure, we suggest that evaluation systems allow gig workers to reject a reasonable proportion of orders without penalty, thereby preserving a degree of job autonomy in task selection and work arrangement. Moreover, the design of evaluative algorithmic evaluation should be further refined to mitigate gig workers’ resistance to this form of evaluation. On the one hand, when algorithmic systems detect minor or recurring performance issues, platforms should provide affected workers with corrective feedback and targeted online training, such as the personalized diagnoses of the factors contributing to delivery delays or low customer ratings. On the other hand, platforms should adopt a transparent and graduated approach based on the severity of each deviation by gig workers, thereby enabling workers to understand the rationale behind any penalties and improve their subsequent work practices.

Third, platforms should carefully calibrate the time pressure imposed through algorithmic control, as high time pressure can intensify the adverse effects of evaluative algorithmic evaluation. To safeguard gig workers’ well-being, algorithmic evaluation systems could first detect sustained periods of intensive work and issue in-app reminders encouraging workers to pause order acceptance and take breaks. In parallel, platforms could allow gig workers to adjust their concurrent order load within predefined limits, thereby giving them greater autonomy to balance earnings and workload according to their individual capacity. Beyond regulating work intensity, platforms should also account for delays caused by factors outside workers’ control. Specifically, evaluation systems could reduce corresponding penalties and adopt dynamic time budgets that adjust task-completion estimates based on real-time conditions, including traffic congestion, adverse weather, restaurant delays, route complexity, and order density. Complementing these measures, platforms could provide customers with updated arrival estimates, explanations for delays, and prompts to remain patient, thereby reducing workers’ concerns that unavoidable delays may result in negative ratings or penalties.

Finally, we offer practical recommendations for promoting gig workers’ thriving at work. As gig platforms become increasingly prominent in labor markets, a growing number of workers are likely to encounter algorithmic evaluation. Approach job crafting enhances gig workers’ thriving at work. Therefore, gig workers are encouraged to use developmental algorithmic evaluation as a job source for work improvement and translate its feedback into opportunities to engage in approach job crafting. Specifically, workers can use feedback generated through developmental algorithmic evaluation to seek advice and support from peers and adjust their work strategies accordingly. Moreover, given that many online gig worker communities are highly active, we encourage gig workers to utilize peer support networks, where they can post questions and solicit advice on how to effectively navigate and leverage algorithmic rules. This enables them to acquire practical knowledge that facilitates approach job crafting.

### 5.4 Limitations and future research

Despite the significant implications of the present study, there are still several limitations which also provide opportunities for future research. First, the sample of this study was limited to food delivery workers in China. Although food delivery represents an important form of gig work, gig work also includes other forms, such as ride-hailing, online freelancing, and other platform-based services. Gig workers’ experiences may differ across different forms of gig work. Therefore, the findings of this study may not be fully generalizable to all types of gig workers. Moreover, because all participants were recruited from China, the cross-cultural generalizability of our findings may be limited. Future research should examine whether similar results can be replicated across different cultural contexts and different types of gig work. Second, all variables in this study were measured using self-reported data collected at a single point in time. Although the statistical tests suggest that common method bias is unlikely to pose a serious threat, the cross-sectional nature of the data may still limit the strength of causal inferences and potential bias may still exist. Therefore, future research could therefore adopt multi-wave survey or experimental designs to further examine the robustness of our findings.

## Data Availability

The datasets presented in this study can be found in online repositories. The names of the repository/repositories and accession number(s) can be found in the article/Supplementary material.
