# Supplementary material for: Developmental or evaluative? Understanding the impact of algorithmic evaluation on gig workers’ thriving at work
Source: Front Psychol. 2026 Aug 18;17:1899280. doi: 10.3389/fpsyg.2026.1899280 (PMC13529468; doi:10.3389/fpsyg.2026.1899280)
Supplement: Supplementary file 1 [file Table_1.docx]

Supplementary Material

# Supplementary Tables-Appendix A

**Table A-1. The Measurement items for this study**

| Constructs | Items | Source |
| --- | --- | --- |
| Developmental algorithmic evaluations | DPA1. I think that the performance evaluation results provided by the platform system can provide me with feedback on my work performance. | (Cleveland et al. 1989) |
|  | DPA2. I think that the performance evaluation results provided by the platform system can identify my strengths and weaknesses. |  |
|  | DPA3. I think that the performance evaluation results provided by the platform system can identify my training needs. |  |
|  | DPA4. I think that the performance evaluation results provided by the platform system can provide me with feedback on areas where I can improve. |  |
| Evaluative algorithmic evaluations | EPA1. I think that the performance evaluation results provided by the platform system determine my compensation. | (Cleveland et al. 1989) |
|  | DPA2. I think that the performance evaluation results provided by the platform system reflect the recognition of my performance. |  |
|  | DPA3. I think that the performance evaluation results provided by the platform system will be used to determine my retention or termination. |  |
| Approach job crafting (Increasing structural resources) | ISTR1. I make an effort to develop myself professionally. | (Harju et al. 2021) |
|  | ISTR2. I make an effort to acquire new things at work. |  |
|  | ISTR3. I make an effort to enhance my capabilities. |  |
| Approach job crafting (Increasing social resources) | ISOR1. I ask customers about their satisfaction with my delivery service. | (Harju et al. 2021) |
|  | ISOR2. I look for inspiration for improvement based on customer feedback. |  |
|  | ISOR3. I seek advice from other delivery workers. |  |
| Approach job crafting (Increasing challenges) | ICH1. When work is less busy, I use the opportunity to plan and start new tasks. | (Harju et al. 2021) |
|  | ICH2. I try to make my work more challenging by examining it from different perspectives. |  |
|  | ICH3. When an interesting task comes along, I proactively offer to be involved. |  |
| Avoidance job crafting | AVJC1. I make an effort to ensure that my work is emotionally less intense | (Harju et al. 2021) |
|  | AVJC2. I make my work so as to minimize interactions with customers who have unrealistic expectations. |  |
|  | AVJC3. I make sure that my work is mentally less intense |  |
| Thriving at work (learning) | LEA1. I often learn new knowledge at work. | (Porath et al. 2012) |
|  | LEA2. I feel that I am continuously improving at work. |  |
|  | LEA3. I feel that I have made a lot of progress at work. |  |
| Thriving at work (vitality) | VIT1. I feel alive and vital at work. | (Porath et al. 2012) |
|  | VIT2. I feel full of energy and spirit at work. |  |
|  | VIT3. I always look forward to each new day at work. |  |
| Time pressure | TIP1. I am always in a hurry to complete my tasks on time. | (Quy Nguyen-Phuoc et al. 2023a) |
|  | TIP2. I frequently think about the penalties for missing deadlines. |  |
|  | TIP3. I frequently worry about missing deadlines due to the time limits on each order. |  |
| Perceived media richness | PMR1. I think social media gives and receives timely feedback timely feedback. | (Gould 1990) |
|  | PMR2. I think social media tailors the messages exchanged. |  |
|  | PMR3. I think social media communicates various cues. |  |
|  | PMR4. I think social media uses rich and varied language. |  |

**Table A-2 Common method bias test**

|  | Factor Loading (R1) | R1² | Method Loading (R2) | R2² |
| --- | --- | --- | --- | --- |
| AVJC_1 | 0.896*** | 0.803 | -0.076** | 0.006 |
| AVJC_2 | 0.917*** | 0.841 | 0.089*** | 0.008 |
| AVJC_3 | 0.934*** | 0.872 | -0.015 | 0.000 |
| DPA_1 | 0.759*** | 0.576 | -0.014 | 0.000 |
| DPA_2 | 0.769*** | 0.591 | 0.006 | 0.000 |
| DPA_3 | 0.790*** | 0.624 | -0.034 | 0.001 |
| DPA_4 | 0.722*** | 0.521 | 0.046 | 0.002 |
| ICH_1 | 0.846*** | 0.716 | -0.032 | 0.001 |
| ICH_2 | 0.832*** | 0.692 | 0.065 | 0.004 |
| ICH_3 | 0.842*** | 0.709 | -0.032 | 0.001 |
| EPA_1 | 0.873*** | 0.762 | -0.030 | 0.001 |
| EPA_2 | 0.843*** | 0.711 | -0.002 | 0.000 |
| EPA_3 | 0.807*** | 0.651 | 0.034 | 0.001 |
| ISOR_1 | 0.863*** | 0.745 | -0.063 | 0.004 |
| ISOR_2 | 0.900*** | 0.810 | 0.045 | 0.002 |
| ISOR_3 | 0.798*** | 0.637 | 0.018 | 0.000 |
| ISTR_1 | 0.777*** | 0.604 | 0.018 | 0.000 |
| ISTR_2 | 0.803*** | 0.645 | 0.067 | 0.004 |
| ISTR_3 | 0.846*** | 0.716 | -0.080 | 0.006 |
| LEA_1 | 0.863*** | 0.745 | -0.103 | 0.011 |
| LEA_2 | 0.854*** | 0.729 | -0.045 | 0.002 |
| LEA_3 | 0.796*** | 0.634 | 0.160** | 0.026 |
| VIT_1 | 0.915*** | 0.837 | -0.057 | 0.003 |
| VIT_2 | 0.911*** | 0.830 | -0.051 | 0.003 |
| VIT_3 | 0.881*** | 0.776 | 0.112* | 0.013 |
| TIP_1 | 0.826*** | 0.682 | -0.045 | 0.002 |
| TIP_2 | 0.859*** | 0.738 | 0.048 | 0.002 |
| TIP_3 | 0.850*** | 0.723 | -0.004 | 0.000 |
| Average |  | 0.711 |  | 0.004 |

Notes: AVJC = avoidance job crafting, DPA = Developmental algorithmic evaluations, ICH = increasing challenges, EPA = evaluative job crafting, ISOR = increasing social resources, ISTR = increasing structural resources, TAW = thriving at work, LEA = learning, VIT = vitality, TIP = time pressure.

**Table A-3. Factor loadings and cross loadings**

|  | AVJC | DPA | ICH | EPA | ISOR | ISTR | LEA | VIT | TIP |
| --- | --- | --- | --- | --- | --- | --- | --- | --- | --- |
| AVJC_1 | ***0.915*** | -0.315 | -0.294 | 0.279 | -0.316 | -0.317 | -0.321 | -0.376 | -0.388 |
| AVJC_2 | ***0.896*** | -0.272 | -0.268 | 0.226 | -0.206 | -0.298 | -0.234 | -0.230 | -0.252 |
| AVJC_3 | ***0.934*** | -0.324 | -0.249 | 0.238 | -0.301 | -0.303 | -0.312 | -0.327 | -0.370 |
| DPA_1 | -0.226 | ***0.742*** | 0.284 | -0.029 | 0.328 | 0.342 | 0.430 | 0.404 | 0.435 |
| DPA_2 | -0.314 | ***0.781*** | 0.394 | -0.004 | 0.372 | 0.371 | 0.416 | 0.331 | 0.388 |
| DPA_3 | -0.264 | ***0.792*** | 0.404 | 0.008 | 0.338 | 0.359 | 0.405 | 0.382 | 0.383 |
| DPA_4 | -0.206 | ***0.723*** | 0.354 | 0.021 | 0.272 | 0.389 | 0.423 | 0.417 | 0.428 |
| ICH_1 | -0.239 | 0.393 | ***0.845*** | -0.033 | 0.358 | 0.463 | 0.408 | 0.366 | 0.409 |
| ICH_2 | -0.291 | 0.442 | ***0.834*** | -0.056 | 0.310 | 0.524 | 0.428 | 0.433 | 0.427 |
| ICH_3 | -0.216 | 0.363 | ***0.841*** | -0.039 | 0.339 | 0.480 | 0.407 | 0.387 | 0.420 |
| EPA_1 | 0.275 | -0.027 | -0.057 | ***0.897*** | -0.056 | -0.018 | -0.086 | -0.097 | -0.050 |
| EPA_2 | 0.189 | 0.029 | -0.032 | ***0.816*** | -0.001 | -0.005 | -0.112 | -0.112 | -0.048 |
| EPA_3 | 0.211 | 0.007 | -0.034 | ***0.804*** | 0.020 | 0.007 | -0.040 | -0.042 | 0.019 |
| ISOR_1 | -0.215 | 0.343 | 0.322 | 0.011 | ***0.855*** | 0.337 | 0.461 | 0.370 | 0.416 |
| ISOR_2 | -0.298 | 0.392 | 0.381 | -0.026 | ***0.903*** | 0.455 | 0.506 | 0.414 | 0.469 |
| ISOR_3 | -0.271 | 0.373 | 0.318 | -0.035 | ***0.803*** | 0.401 | 0.423 | 0.345 | 0.388 |
| ISTR_1 | -0.250 | 0.377 | 0.437 | 0.023 | 0.375 | ***0.775*** | 0.414 | 0.398 | 0.440 |
| ISTR_2 | -0.285 | 0.405 | 0.501 | -0.040 | 0.407 | ***0.812*** | 0.441 | 0.429 | 0.436 |
| ISTR_3 | -0.277 | 0.383 | 0.472 | 0.001 | 0.352 | ***0.838*** | 0.411 | 0.387 | 0.412 |
| LEA_1 | -0.317 | 0.399 | 0.368 | -0.107 | 0.478 | 0.390 | ***0.858*** | 0.547 | 0.543 |
| LEA_2 | -0.255 | 0.453 | 0.403 | -0.078 | 0.463 | 0.426 | ***0.852*** | 0.563 | 0.554 |
| LEA_3 | -0.236 | 0.529 | 0.471 | -0.049 | 0.425 | 0.498 | ***0.804*** | 0.574 | 0.542 |
| VIT_1 | -0.302 | 0.462 | 0.426 | -0.074 | 0.348 | 0.463 | 0.573 | ***0.912*** | 0.507 |
| VIT_2 | -0.314 | 0.397 | 0.409 | -0.087 | 0.432 | 0.440 | 0.587 | ***0.909*** | 0.507 |
| VIT_3 | -0.330 | 0.498 | 0.439 | -0.108 | 0.414 | 0.453 | 0.651 | ***0.885*** | 0.537 |
| TIP_1 | -0.268 | 0.445 | 0.410 | 0.022 | 0.445 | 0.401 | 0.571 | 0.403 | ***0.802*** |
| TIP_2 | -0.368 | 0.474 | 0.445 | -0.044 | 0.399 | 0.480 | 0.562 | 0.513 | ***0.882*** |
| TIP_3 | -0.307 | 0.436 | 0.406 | -0.053 | 0.430 | 0.458 | 0.526 | 0.526 | ***0.846*** |

**Table A-4. The weights of formative construct**

| Construct | Indicator | Weight | VIF |
| --- | --- | --- | --- |
| Approach job crafting | Increasing structural resources | 0.400*** | 1.679 |
|  | Increasing social resources | 0.458*** | 1.321 |
|  | Increasing challenges | 0.379*** | 1.562 |
| Thriving at work | Learning | 0.669*** | 1.813 |
|  | Vitality | 0.420*** | 1.813 |

Note: ****p* <.001.

# Data Availability Statement

The datasets for this study can be found in *OSF* project <https://osf.io/Z37n2/>
